# The role of immune cell signatures in the pathogenesis of ovarian-related diseases: a causal inference based on Mendelian randomization

**DOI:** 10.1097/JS9.0000000000001814

**Published:** 2024-06-17

**Authors:** Yangguang Lu, Yingyu Yao, Sijia Zhai, Feitian Ni, Jingyi Wang, Feng Chen, Yige Zhang, Haoyang Li, Hantao Hu, Hongzhi Zhang, Bohuai Yu, Hongbo Chen, Xianfeng Huang, Weiguo Ding, Di Lu

**Affiliations:** a The First School of Medicine, School of Information and Engineering; b The Second School of Medicine, Wenzhou Medical University, Wenzhou; c Tongde Hospital of Zhejiang Province; d The Second Affiliated College, Zhejiang Chinese Medical University, Hangzhou; e School of Acupuncture-Moxibustion and Tuina, School of Health Preservation and Rehabilitation, Nanjing University of Chinese Medicine, Nanjing, People’s Republic of China

**Keywords:** genetic analyses, immune cells, immunology, Mendelian randomization, ovarian tumor, ovarian-related diseases

## Abstract

**Background:**

Immune cells play a pivotal role in maintaining ovarian function. However, the specific contributions of different immune cell phenotypes to the pathogenesis of specific ovarian-related diseases remain poorly understood. The authors aim to investigate the correlation between 731 immunophenotypes and ovarian-related diseases.

**Materials and methods:**

Utilizing publicly available genetic data, the authors undertook a series of quality control measures to identify instrumental variables associated with exposure. Subsequently, we conducted two-sample Mendelian randomization (MR) using inverse variance weighting to explore the causal relationships between 731 immune cell features and six ovarian-related diseases: ovarian cysts, ovarian dysfunction, premature ovarian failure (POF), polycystic ovary syndrome (PCOS), benign neoplasm of ovary, and malignant neoplasm of ovary at the genetic level. Sensitivity analyses, including leave-one-out and other MR analysis models, were performed. Finally, Bayesian colocalization (COLOC) analysis was employed to identify specific co-localized genes, thereby validating the MR results.

**Results:**

At the significance level corrected by Bonferroni, four immune phenotypes, including CD25 on IgD- CD38- B cells, were associated with ovarian cysts; four immune phenotypes, including CD39+ CD4+ T cell Absolute Count, were associated with ovarian dysfunction; eight immune phenotypes, including SSC-A on HLA DR+ CD8+ T cells, were associated with POF; five immune phenotypes, including CD20- CD38- B cell Absolute Count, were associated with PCOS; five immune phenotypes, including CD4+ CD8dim T cell Absolute Count, were associated with benign ovarian tumors; and three immune phenotypes, including BAFF-R on IgD- CD38+ B cells, were associated with malignant ovarian tumors. Sensitivity analysis indicated robust results. COLOC analysis identified four immune cell co-localized variants (rs150386792, rs117936291, rs75926368, and rs575687159) with ovarian diseases.

**Conclusion:**

Our study elucidates the close genetic associations between immune cells and six ovarian-related diseases, thereby providing valuable insights for future research endeavors and clinical applications.

## Graphical abstract

**Figure:**
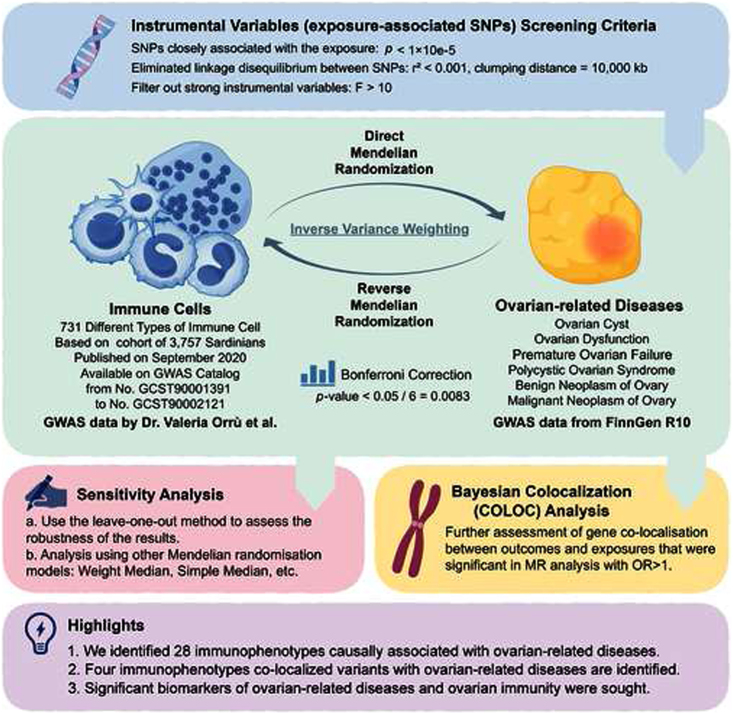

## Introduction

Highlights

- We identified 28 immunophenotypes causally associated with ovarian-related diseases.
- Four immunophenotypes co-localized variants with ovarian-related diseases are identified.
- Significant biomarkers of ovarian-related diseases and ovarian immunity were sought.

Immune cells play pivotal roles in the developmental processes of the ovary^1^. Animal studies have revealed that macrophages are the most abundant immune cell population in the adult mouse ovary, widely distributed in the theca, stroma, and corpus luteum regions^2,3^. In human ovaries, a macrophage population predominantly expressing CD68 is situated near vascular cells and the luteal cell layer of the corpus luteum^4,5^. As the most versatile cells within the immune system, the homeostasis of macrophages is closely linked to pathological changes such as pregnancy complications and ovarian cancer^6,7^. Additionally, variations in the levels of lymphocytes, including T cells, B cells, and natural killer (NK) cells, which primarily function in adaptive immune responses, may also correlate with the progression and prognosis of ovarian cancer^8^. Apart from ovarian tumors, past studies have demonstrated significant causal relationships between immune cells and other ovarian disorders such as premature ovarian failure (POF) and polycystic ovary syndrome (PCOS)^9,10^.

Numerous ovarian-related disorders pose threats to global female reproductive function and fertility, with unclear etiologies^11,12^. Among these, PCOS has emerged as a major international public health concern. Particularly in China, 7.8% of reproductive-age women suffer from PCOS, with a significant increase in prevalence observed over the past decade^13^. Furthermore, epidemiological screenings indicate that ~5–10% of women with secondary amenorrhea have POF^14^, while up to 18% of postmenopausal women develop simple ovarian cysts^15^. The impact and challenges posed by such conditions on women’s health are evident. Therefore, further exploration of the pathogenic mechanisms of these disorders, especially elucidating the role of immune cells in ovarian-related diseases, is imperative. However, considering that prior research has primarily focused on the role of macrophage populations, there is limited knowledge regarding the involvement of other types of immune cells such as monocytes and mast cells in ovarian physiological functions^1,16^, necessitating broader investigations into the roles played by various immune cell types and their characteristics in the pathogenesis of ovarian-related diseases.

Mendelian randomization (MR) is an epidemiological causal inference method based on Mendel’s laws of independent assortment, widely employed in investigating the etiology of various diseases^17^. This approach minimizes confounding factors and reverse causation biases in epidemiological research, as genetic variations precede disease onset^18^. In this study, we comprehensively explore the causal relationships based on genetic levels between 731 immune cell traits and six ovarian-related diseases, including ovarian cysts, ovarian dysfunction, POF, PCOS, benign neoplasm of ovary, and malignant neoplasm of ovary, using MR. This research aims to identify new targets for the prediction and treatment of ovarian-related diseases.

## Materials and methods

### Study design

This study adhered to the Strengthening the Reporting of Observational Studies in Epidemiology Using Mendelian Randomization (STROBE-MR) guidelines^18^. We utilized summary data from published genome-wide association studies (GWAS) encompassing 731 immune cell traits and six distinct ovarian-related diseases, selecting appropriate single nucleotide polymorphisms (SNPs) as instrumental variables (IVs) for MR analysis to investigate their bidirectional causal relationships. Additionally, we conducted Bayesian colocalization (COLOC) analysis to identify SNP loci simultaneously acting on immune cells and ovarian diseases^19^. All data for this study were sourced from previously published research and public databases, thus obviating the need for additional ethical approval.

### GWAS data sources

Data on immune cells were derived from a GWAS study conducted by Orrù *et al*.^20^, involving a cohort of 3757 individuals from Sardinia (Table 1). The study encompassed 731 immune phenotypes, including 118 absolute cell counts (AC), 389 median fluorescence intensity (MFI) reflecting surface antigen levels, 32 morphological parameters (MP), and 192 relative cell counts (RC). Among these, MFI, AC, and RC features comprised B cells, CDCs, mature stage T cells, monocytes, marrow cells, TBNK (T cells, B cells, and NK cells), and Treg cells, while MP features included CDC and TBNK cells. Comprehensive details of the study procedures can be found in the published research. Summary statistics for each immune trait GWAS are publicly available from the GWAS Catalog (registration numbers GCST90001391 to GCST90002121).

**Table 1:** Information on the GWAS data cohort used to conduct the MR analysis.

Data source	Population	Phenotype	Sample size	No. of cases	No. of controls
FinnGen R10	European (Finnish)	Ovarian cyst	134 466	22 883	111 583
		Ovarian dysfunction	221 279	2309	218 970
		Premature ovarian failure	219 512	542	218 970
		Polycystic ovarian syndrome	220 609	1639	218 970
		Benign neoplasm of ovary	230 310	5732	224 578
		Malignant neoplasm of ovary	184 018	1091	182 927
Orrù et al. (2020)^20^	European (Sardinian)	731 Immunophenotypes (AC/MFI/MP/RC)	3757	N.A.	N.A.

Note. AC, absolute cell counts; MFI, median fluorescence intensity; MP, morphological parameters; RC, relative cell counts.

Genetic data for six ovarian-related diseases, including ovarian cysts, ovarian dysfunction, POF, PCOS, benign neoplasm of ovary, and malignant neoplasm of ovary, were sourced from the FinnGen consortium (https://r10.finngen.fi/, accessed on 28 January 2024). The study cohorts comprised European ancestry individuals who provided informed consent. The FinnGen research project integrates genetic data related to disease endpoints from the Finnish Biobank and the Finnish National Registry (Table 1). Case identification was based on International Classification of Diseases, Tenth Revision (ICD-10) codes. Detailed information regarding participant characteristics, genetic typing, imputation, and quality control can be found on the FinnGen website (https://finngen.gitbook). Importantly, there was no overlap between two GWAS cohort, enabling the possibility of conducting two-sample MR studies.

### Instrumental variable selection

To ensure the robustness and reliability of MR analysis, we applied the following criteria for IV selection. The application of IVs in MR analysis relies on meeting three key assumptions: (i) the selected IVs exhibit strong associations with the exposure of interest, (ii) IVs are not confounded by factors influencing the outcome other than the exposure, and (iii) the selected IVs affect the outcome solely through the exposure^21^. To enable SNPs to be associated with the exposure, we set the significance threshold for IVs at 1×10^−5^. Furthermore, we eliminated linkage disequilibrium (LD) between SNPs, as strong LD can introduce bias (r²<0.001, clumping distance = 10 000 kb), selecting the variant with the lowest *P*-value associated with the exposure in the presence of LD genetic variation. Subsequently, we filtered out weak IVs (F>10) to ensure a strong correlation between IVs and exposure. Finally, we harmonized SNPs for exposure and outcome to ensure consistent effect estimates for the same effect allele and excluded palindromic SNPs or SNPs with incompatible effect allele frequencies.

### Statistical analysis

We employed the inverse variance-weighted (IVW) method to assess the correlation between exposure and outcome^22^. When all IVs satisfy three crucial assumptions, the IVW method provides accurate and stable estimates. We presented our results in the form of β values and their standard errors (SE) or odds ratios (ORs) and their 95% CI. We used Cochrane’s *Q* test to measure heterogeneity and quantified it using the *I*² statistic. Heterogeneity was deemed absent when *I*² was less than 25% and mild when *I*² was less than 50%. We evaluated potential horizontal pleiotropy using the intercept from MR-Egger regression and the MR pleiotropy residual sum and outlier (MR-PRESSO)^21,23^. To ensure the robustness of the results, we conducted sensitivity analyses using the leave-one-out method to identify SNPs that may have potentially influential effects. Furthermore, for outcomes with *P*<0.10 in previous MR analyses, we used alternative MR analysis models as supplementary methods to validate the significance of the conclusions, including the MR-Egger method, Weighted Median method, and Simple Median method. The MR-Egger regression adjusts for potential horizontal pleiotropy by sacrificing estimation precision^21^, whereas the weighted median method provides accurate estimates by assuming that at least 50% of IVs are valid^24^. For immune phenotypes and ovarian diseases identified as having causal relationships in MR analysis, we further conducted reverse MR analysis to infer bidirectional causality.

To better validate the results of MR analysis and explore the co-localized effects of specific SNP loci and genes driving immune phenotypes on the risk of ovarian-related diseases in the same direction as identified in forward MR analysis with causal relationships and OR>1, we conducted COLOC analysis on GWAS data for ovarian diseases and immune cells. COLOC analysis is a statistical method based on GWAS data that employs univariate summary statistics to assess whether two independently correlated genetic traits share common genetic loci. In this analysis, we assigned a prior probability of 1×10^−6^ to the random variables causally linked to both GWAS datasets. This value indicates a sufficiently high posterior probability (PP4>0.8) that immune phenotypes and ovarian-related diseases share a single common variant^19^. For the identified co-localized SNPs, we searched for their minor allele frequencies (MAF) in European populations in the gnomAD v4.0.0 database to assess the rarity of the locus. MAF<0.01 was defined as a rare mutation, and MAF<0.001 was defined as an ultra-rare mutation.

In MR analysis, considering that each immune phenotype exposure corresponds to outcomes of six ovarian-related diseases, we applied Bonferroni correction for multiple testing to significance levels, and *P*<0.0083 was considered statistically significant (0.05 divided by 6). In tests for heterogeneity and horizontal pleiotropy, *P*<0.05 was considered statistically significant. All statistical analyses were conducted using R version 4.3.2.

## Results

### Causal effect between immunophenotypes and ovarian cyst

**Figure 1:**
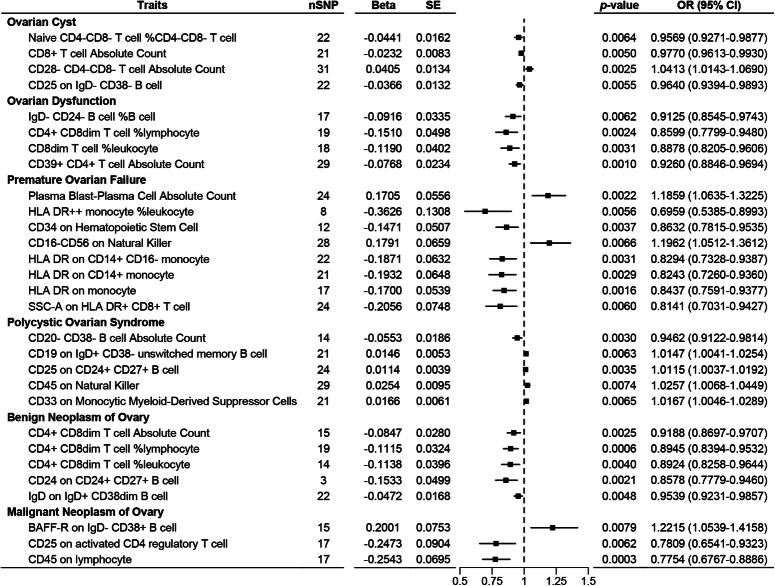
Forest plot of positive results in Mendelian randomization analysis of the role of immunophenotypes on ovarian-related diseases.

**Figure 2:**
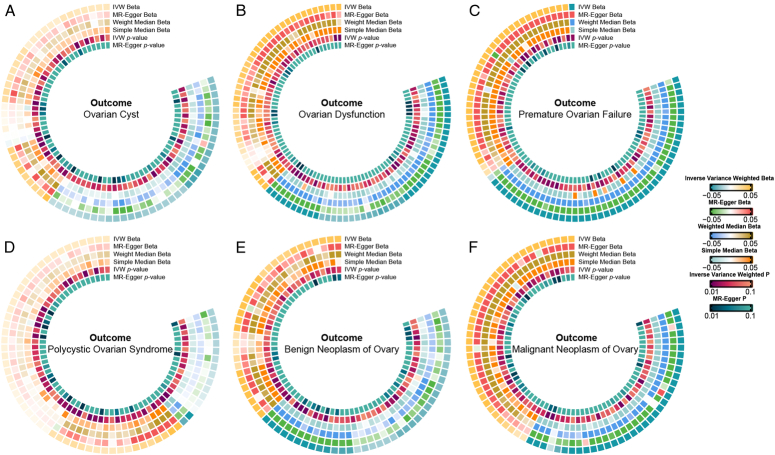
Heat map of the sensitivity analysis carried out by applying various MR analysis models. (A) IgD- CD24- B cell AC and 72 other immunophenotypes with *P*<0.01 on ovarian cyst; (B) Memory B cell AC and 90 other immunophenotypes with *P*<0.01 on ovarian dysfunction; (C) Plasma Blast-Plasma Cell AC and 83 other immunophenotypes with *P*<0.01 on POF; (D) IgD+ CD38+ B cell AC and 68 other immunophenotypes with *P*<0.01 on PCOS; (E) Switched memory B cell %B cell and 70 other immunophenotypes with *P*<0.01 on benign neoplasm of ovary; (F) Switched memory B cell AC and 74 other immunophenotypes with *P*<0.01 on malignant neoplasm of ovary.

**Figure 3:**
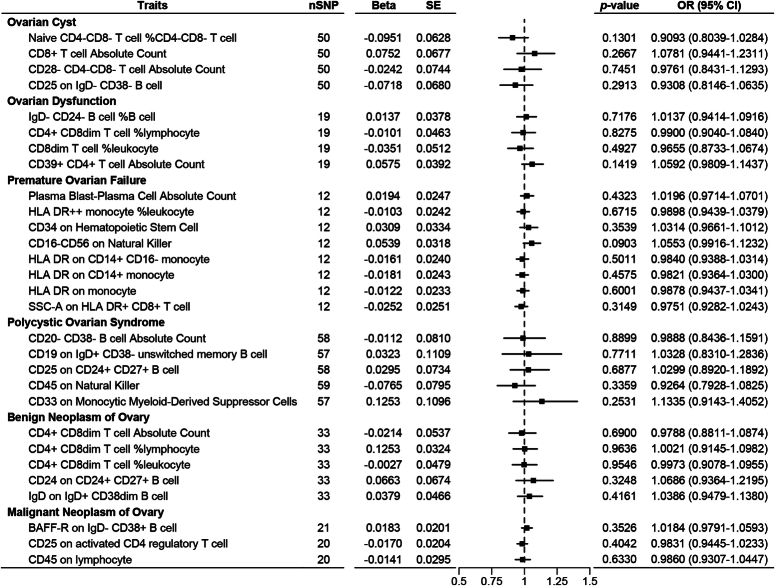
Forest plot of the reverse Mendelian randomization analysis carried out on immunophenotypes and ovarian-related diseases with direct causality.

Five immune phenotypes showed associations with ovarian cysts. Results from the IVW model revealed that CD28-CD4-CD8- T cell AC was positively associated with an increased risk of ovarian cysts (β=0.0405±0.0083, *P*=0.0025), while Naive CD4-CD8- T cell %CD4-CD8- T cell (β =−0.0441±0.0162, *P*=0.0064), CD8+ T cell AC (β =−0.0232±0.0083, *P*=0.0050), and CD25 on IgD-CD38- B cell (β =−0.0366±0.0132, *P*=0.0055) were negatively associated with the risk of ovarian cysts (Fig. 1). There was no significant horizontal pleiotropy among IVs, although mild heterogeneity was observed in the MR analysis with CD25 on IgD-CD38- B cell as the exposure (*I*²=47.46%, *P*=0.0120) (Table 2). Sensitivity analysis using the leave-one-out method indicated robust results, with no changes in the effect size and significance of immune phenotypes after switching to other MR models (Fig. 2A). No causal effect of ovarian cysts on these five immune phenotypes was found in the reverse MR analysis (Fig. 3).

**Table 2:** Heterogeneity and horizontal pleiotropy of positive results of MR analysis with immune cells as exposure and ovarian-related disease as outcome.

	Heterogeneity		Pleiotropy		
Traits	*I* ^2^	*P*	Egger intercept	SE	*P*
a. Ovarian Cyst as outcome.					
Naive CD4-CD8- T cell %CD4-CD8- T cell	11.23	0.3693	0.0003	0.0049	0.9554
CD8+ T cell Absolute Count	22.72	0.1775	−0.0038	0.0042	0.3742
CD28- CD4-CD8- T cell Absolute Count	7.21	0.4020	−0.0007	0.0048	0.8930
CD25 on IgD- CD38- B cell	47.46	0.0120	0.0020	0.0062	0.7465
b. Ovarian Dysfunction as outcome.					
IgD- CD24- B cell %B cell	13.11	0.5878	0.0107	0.0170	0.5401
CD4+ CD8dim T cell %lymphocyte	0.00	0.7298	0.0346	0.0188	0.0835
CD8dim T cell %leukocyte	10.16	0.3352	−0.0057	0.0199	0.7785
CD39+ CD4+ T cell Absolute Count	7.02	0.5616	−0.0232	0.0119	0.0606
c. Premature Ovarian Failure as outcome.					
Plasma Blast-Plasma Cell absolute count	35.86	0.0458	−0.0224	0.0299	0.4614
HLA DR++ monocyte %leukocyte	0.00	0.7210	−0.0479	0.0628	0.4801
CD34 on hematopoietic stem cell	17.65	0.2755	−0.0310	0.0401	0.4573
CD16-CD56 on natural killer	8.93	0.5834	0.0114	0.0460	0.8058
HLA DR on CD14+ CD16- monocyte	0.00	0.8748	0.0672	0.0437	0.1406
HLA DR on CD14+ monocyte	0.00	0.7633	0.0653	0.0441	0.1563
HLA DR on monocyte	0.00	0.6347	0.0132	0.0421	0.7589
SSC-A on HLA DR+ CD8+ T cell	0.00	0.7768	−0.0175	0.0295	0.5597
d. Polycystic Ovarian Syndrome as outcome.					
CD20- CD38- B cell absolute count	5.01	0.4967	−0.0064	0.0069	0.3703
CD19 on IgD+ CD38- unswitched memory B cell	0.00	0.6213	−0.0094	0.0059	0.1268
CD25 on CD24+ CD27+ B cell	13.90	0.2683	−0.0039	0.0030	0.2085
CD45 on natural killer	3.43	0.5143	0.0016	0.0036	0.6529
CD33 on monocytic myeloid-derived suppressor cells	4.07	0.4061	0.0020	0.0038	0.5965
e. Benign Neoplasm of Ovary as outcome.					
CD4+ CD8dim T cell Absolute Count	0.00	0.8635	−0.0035	0.0117	0.7667
CD4+ CD8dim T cell %lymphocyte	2.49	0.4848	0.0068	0.0123	0.5903
CD4+ CD8dim T cell %leukocyte	11.50	0.5558	−0.0111	0.0163	0.5083
CD24 on CD24+ CD27+ B cell	33.75	0.3872	0.0018	0.0231	0.9498
IgD on IgD+ CD38dim B cell	0.00	0.6962	0.0065	0.0091	0.4820
f. Malignant Neoplasm of Ovary as outcome.					
BAFF-R on IgD- CD38+ B cell	0.00	0.7758	0.0261	0.0322	0.4319
CD25 on activated CD4 regulatory T cell	6.78	0.5258	0.0383	0.0394	0.3464
CD45 on lymphocyte	7.79	0.3632	0.0474	0.0461	0.3196

Note. SE, Standard error.

### Causal effect between immunophenotypes and ovarian dysfunction

Four immune phenotypes were associated with ovarian dysfunction. Results from the IVW model showed that IgD-CD24- B cell %B cell (β =−0.0916±0.0335, *P*=0.0062), CD4+ CD8dim T cell %lymphocyte (β =−0.1510±0.0498, *P*=0.0024), CD8dim T cell %leukocyte (β=−0.1190±0.0402, *P*=0.0031), and CD39+ CD4+ T cell AC (β=0.0768±0.0234, *P*=0.0010) were negatively associated with the risk of ovarian dysfunction (Fig. 1). There was no significant heterogeneity or horizontal pleiotropy among IVs (Table 2). Sensitivity analysis indicated robust results, with no changes in the effect size and significance of immune phenotypes after switching to other MR models (Fig. 2B). No causal effect of ovarian dysfunction on these four immune phenotypes was found in the reverse MR analysis (Fig. 3).

### Causal effect of immunophenotypes on POF

Eight immune phenotypes were associated with POF. Results from the IVW model indicated that Plasma Blast-Plasma Cell AC (β=0.1705±0.0556, *P*=0.0022) and CD16-CD56 on NK (β=0.1791±0.0659, *P*=0.0066) were positively associated with an increased risk of POF, while HLA DR++ monocyte %leukocyte (β =−0.3626±0.1308, *P*=0.0056), CD34 on Hematopoietic Stem Cell (β =−0.1471±0.0507, *P*=0.0037), HLA DR on CD14+ monocyte (β=−0.1871±0.0632, *P*=0.0031), HLA DR on CD14+ monocyte (β =−0.1932±0.0648, *P*=0.0029), HLA DR on monocyte (β=−0.1700±0.0539, *P*=0.0016), and SSC-A on HLA DR+ CD8+ T cell (β =−0.2056±0.0748, *P*=0.0060) were negatively associated with the risk of POF (Fig. 1). There was no significant horizontal pleiotropy among IVs, although mild heterogeneity was observed in the MR analysis with Plasma Blast-Plasma Cell AC as the exposure (*I*²=35.86%, *P*=0.0458) (Table 2). Sensitivity analysis indicated robust results, with no changes in the effect size and significance of immune phenotypes after switching to other MR models (Fig. 2C). No causal effect of POF on these eight immune phenotypes was found in the reverse MR analysis (Fig. 3).

### Causal effect of immunophenotypes on PCOS

Five immune phenotypes were associated with PCOS. Results from the IVW model showed that CD19 on IgD+ CD38- unswitched memory B cell (β =0.0146±0.0053, *P*=0.0063), CD25 on CD24+ CD27+ B cell (β=0.0114±0.0039, *P*=0.0035), CD45 on NK (β=0.0254±0.0095, *P*=0.0074), and CD33 on Monocytic Myeloid-Derived Suppressor Cells (β=0.0166±0.0061, *P*=0.0065) were positively associated with an increased risk of PCOS, while CD20- CD38- B cell AC was negatively associated with the risk of PCOS (β=−0.0553±0.0186, *P*=0.0030) (Fig. 1). There was no significant heterogeneity or horizontal pleiotropy among IVs (Table 2). Sensitivity analysis indicated robust results, with no changes in the effect size and significance of immune phenotypes after switching to other MR models (Fig. 2D). No causal effect of PCOS on these five immune phenotypes was found in the reverse MR analysis (Fig. 3).

### Causal effect of immunophenotypes on neoplasm of ovary

There were eight immune phenotypes associated with ovarian tumors. Among them, CD4+ CD8dim T cell AC (β=−0.0847±0.0280, *P*=0.0025), CD4+ CD8dim T cell %lymphocyte (β=−0.1115±0.0324, *P*=0.0006), CD4+ CD8dim T cell %leukocyte (β=−0.1138±0.0396, *P*=0.0040), CD24 on CD24+ CD27+ B cell (β=−0.1533±0.0499, *P*=0.0021), and IgD on IgD+ CD38dim B cell (β=−0.0472±0.0168, *P*=0.0048) were associated with a decreased risk of Benign Neoplasm of Ovary (Fig. 1). Sensitivity analysis using the leave-one-out method and other MR models indicated robust results (Fig. 2E). Additionally, BAFF-R on IgD- CD38+ B cell (β=0.2011±0.0753, *P*=0.0079) was associated with an increased risk of malignant neoplasm, while CD25 on activated CD4 regulatory T cell (β=−0.2473±0.0904, *P*=0.0062) and CD45 on lymphocyte (β=−0.2543±0.0695, *P*=0.0003) were associated with a decreased risk of malignant neoplasm (Fig. 1). There was no significant heterogeneity or horizontal pleiotropy among IVs, although mild heterogeneity was observed in the MR analysis with CD24 on CD24+ CD27+ B cell as the exposure and benign neoplasm of ovary as the outcome (*I*²=33.75%, *P*=0.3872) (Table 2). Sensitivity analysis using the leave-one-out method and other MR models indicated robust results (Fig. 2F). No causal effect of ovarian malignant or benign neoplasm on these eight immune phenotypes was found in the reverse MR analysis (Fig. 3).

### Bayesian colocalization between immunophenotypes and ovarian-related diseases

In the previous forward MR analysis, ORs for eight entries were greater than 1. Through COLOC analysis pairing exposure and outcome GWAS for these eight entries, four colocalized SNP loci were identified (Table 3). Among them, CD28- CD4-CD8- T cell AC colocalized with ovarian cysts on rs150386792 (PP4=0.9549), although this SNP was not located on a gene locus. CD16-CD56 on NK colocalized with POF on rs117936291 (PP4=0.9988) and rs75926368 (PP4=0.8760), where rs117936291 was located on the NXPE2 gene locus and rs75926368 on the LINC02762 gene locus. Additionally, CD19 on IgD+ CD38- unswitched memory B cell colocalized with PCOS on rs575687159 (PP4=0.9854), located on the *PLXNA2* gene locus. By searching for these four loci in the gnomAD database to determine their MAF in all populations, a mutation was identified in rs575687159 on chromosome 1 (MAF=0.0019) and in rs150386792 on chromosome 6 (MAF=0.0046) as rare mutations. None of these four mutations were classified as ultra-rare.

**Table 3:** COLOC gene co-localization analysis for items with OR >1 in the significance results of MR analysis.

SNP	Position	MAF	Allele	Gene	PP4
a. CD28- CD4-CD8- T cell absolute count on ovarian cyst					
rs150386792	chr6:97780970	0.0046	G>A	N.A.	0.9549
b. CD16-CD56 on natural killer on premature ovarian failure					
rs117936291	chr11:114492374	0.0290	A>G	*NXPE2*	0.9988
rs75926368	chr11:112287601	0.0114	T>A	*LINC02762*	0.8760
c. CD19 on IgD+ CD38- unswitched memory B cell on polycystic ovarian syndrome					
rs575687159	chr1:208096600	0.0019	C>T	*PLXNA2*	0.9854

Note. MAF, Minor allele frequency (based on gnomAD v4.0.0); HGVS, Human Genome Variation Society nomenclature.

## Discussion

Drawing from extensive publicly available GWAS data, we investigated the causal relationships between 731 immune cell phenotypes and six ovarian-related diseases at the genetic level. To our knowledge, this represents the first MR and COLOC analysis to explore the causal links between multiple immune phenotypes and various ovarian-related diseases. Our study identified 28 immune phenotypes positively associated with ovarian-related diseases.

T cells play a pivotal role in cellular immune processes. Our MR analysis results suggest that elevated levels of CD4+ CD8dim T cells act as protective factors against ovarian dysfunction and benign neoplasm of ovary. CD4+ CD8dim T cells, a subset of lymphocytes expressing both CD4 and CD8 molecules, are known to be critical in antitumor and autoimmune responses^25,26^. Our findings further affirm that CD4+ CD8dim T cells may safeguard ovarian function and structure by maintaining immune system equilibrium. Their abundance or proportion among lymphocytes and leukocytes can serve as sensitive indicators of the body’s immune status and tumor resistance levels. Decreases in CD4+ CD8dim T cell levels may serve as sensitive predictive indicators for benign neoplasm of ovary. Additionally, the onset of ovarian dysfunction may correlate with reductions in various T cell levels, such as CD8dim T cells and CD39+ CD4+ T cells. Prior research has hinted at a link between heightened immunosuppressive regulatory T cells and adverse outcomes in ovarian malignancies^8^. However, our MR analysis reveals that increased CD25 expression on activated CD4 regulatory T cells has a protective effect against ovarian malignancies, thereby supplementing previous conclusions.

Significantly, we also observed negative correlations between several monocyte phenotypes, including HLA DR on CD14+ CD16- monocytes, HLA DR on CD14+ monocytes, and HLA DR on monocytes, and the risk of POF. HLA-DR is a major histocompatibility complex (MHC) II molecule whose surface expression on monocytes reflects their activation status^27^, aiding in immune status assessment^28^. The three monocyte subpopulations identified in our study may possess immunosuppressive properties by inhibiting T cell proliferation and differentiation, fostering regulatory T cell generation, and secreting anti-inflammatory factors, thereby shielding the ovaries from autoimmune damage and reducing the risk of POF. Additionally, we noted that an increase in Plasma Blast-Plasma Cell AC is a risk factor for POF. This could denote excessive B cell activation and differentiation, leading to autoantibody production and autoimmune reactions^29^. Autoantibodies may cross-react with ovarian tissue antigens, triggering inflammation and ovarian damage. Hence, the level of Plasma Blast-Plasma Cell could serve as a clinical predictor for POF.

The causal association between immune cell phenotypes and PCOS was a central focus of our study. MR analysis suggested associations between PCOS and various B cell immune phenotypes. We discovered that elevated levels of CD20- CD38- B cells exert a protective effect against PCOS occurrence. CD20, a specific antigen located on B lymphocyte surfaces, likely plays a crucial role in regulating B lymphocyte proliferation, differentiation, and signal transduction processes^30^. Therefore, CD20- CD38- B cells may safeguard normal ovarian morphology by preserving normal immune system pathways. Additionally, memory B cells are rich in autoantibodies and are associated with chronic infections, autoimmune diseases, and the accumulation of diseases. Prior studies have indicated significant B cell repertoire recombination and increased B memory cell frequency in hyperandrogenic PCOS patients^31^. Our findings confirm that the elevation of CD19 on IgD+ CD38- unswitched memory B cells may contribute to PCOS occurrence. Finally, researchers have observed a decrease in CD2 surface density on peripheral blood NK cells in a mouse model of PCOS^32^. However, we did not observe this in our MR study of the European population; instead, an increase in CD45 surface density on NK cells may be a risk factor for PCOS occurrence. The specific pathogenic effects of NK cells and their immune phenotypes in PCOS require validation through future clinical observational studies.

Previous researchers have utilized the MR method to investigate the correlation between immune cells and PCOS^10^. However, this study did not employ reverse MR to uncover bidirectional causality or conduct COLOC analysis to identify co-localized acting genes. In our investigation, COLOC analysis focused on the co-localization of CD19 on IgD+ CD38- unswitched memory B cells and PCOS on the *PLXNA2* gene. This observation has not been previously reported by researchers. The *PLXNA2* gene encodes plexin A2 protein, a signaling co-receptor involved in neuronal system development and axon guidance signal transduction^33,34^. Signaling molecules play critical roles in various stages of physiological and pathological immune responses, regulating immune cell activation, differentiation, or guiding immune cell trafficking^35^. Mutations related to this gene may trigger overreactions in the neuroendocrine system, stimulating the proliferation and differentiation of corresponding memory B cells. This could lead to oxidative stress, inflammation, or fibrosis in the ovaries, culminating in PCOS occurrence^36^. Another plausible explanation is that the expression level of *PLXNA2* impacts the formation of vascular endothelial cells^37^, which are closely linked to ovarian development and immunity^1^. Furthermore, through COLOC analysis, we identified the co-localization of CD16-CD56 on NK cells with POF on genes *NXPE2* and *LINC02762*. The specific function of *NXPE2* is unclear, but previous research suggests a connection between *NXPE2* protein and inflammatory diseases such as ulcerative colitis, inflammatory bowel disease, and Crohn’s disease^38–40^, indicating the need for more evidence to elucidate its effects on NK cells and ovarian function. The transcript product of *LINC02762* is a long-chain noncoding RNA that may play a regulatory role in the expression of immune-related proteins.

Our study was based on published large-scale GWAS studies, including a substantial sample of ~200 000 individuals, providing considerable statistical power. Additionally, we employed Bonferroni correction for significance and conducted comprehensive sensitivity analyses to ensure the robustness of the results. However, it is crucial to acknowledge the significant inherent limitations of this study. Firstly, large-scale GWAS studies on immune cells are currently limited to European populations, so our conclusions are ethnically constrained. Considering the genetic diversity of different racial groups, the relationship between immune cells and ovarian-related diseases we established may vary in non-European populations. Secondly, we must note that MR analysis and COLOC co-localization analysis are solely based on observations at the genetic level and causal inference, and cannot replace objective clinical trials in the field. Finally, due to the limitations of the MR study method, the timing of immune cell actions in the occurrence of ovarian diseases may be overlooked, and potential confounding factors may not be fully addressed. Therefore, we urge researchers worldwide to consider the findings of our preliminary study and conduct multicenter clinical cohort studies to further understand the cause-effect relationship between immunity and ovarian diseases.

Our study has significant clinical implications. Firstly, our research can offer new biomarkers for the early diagnosis and prevention of ovarian-related diseases. For instance, levels of CD28- CD4-CD8- T cells, Plasma Blast-Plasma Cell, or CD20- CD38- B cells could be utilized to predict the occurrence of ovarian cysts, ovarian dysfunction, and PCOS. Subsequent researchers can further explore the sensitivity and specificity of their abundance or ratios among lymphocytes and leukocytes in predicting relevant ovarian diseases. Secondly, our study can provide new targets and strategies for immune therapy of ovarian-related diseases. Additionally, our research can offer new insights into the pathogenesis and genetic factors of ovarian-related diseases, such as the reciprocal regulation between immune cells and ovaries. Finally, our study can serve as an initial exploration into ovarian disease-immune cell interactions for future researchers. For example, immune cells may act as intermediate factors in the pathogenesis of ovarian-related diseases, and future studies can conduct large-sample, multicenter cohort studies to further explore the mediating role of immune phenotypes in various risk factors for ovarian-related diseases.

## Conclusion

In summary, through comprehensive MR and COLOC analysis, we have unveiled complex causal relationships at the genetic level between 731 immune phenotypes and six ovarian-related diseases, identifying 28 immune phenotypes and 4 co-localized SNPs involved in the pathogenesis of ovarian diseases. We emphasize the intricate pattern of interaction between the immune system and ovarian function. Our study provides new biomarkers and treatment targets for the clinical management of preventing and treating ovarian-related diseases, while also opening up new avenues for researchers to explore the relationship between immunity and ovaries. However, the etiology of ovarian diseases is multifactorial, and various types of immune cells exhibit apparent immunosuppressive effects. Therefore, further clinical observations considering comprehensive factors are necessary to better elucidate the roles of innate and adaptive immune cells.

## Ethical approval

Only publicly available data were used for this study. Ethical approval for all data used can be found in the original publication.

## Consent

Not applicable.

## Source of funding

The author(s) declare financial support was received for the research, authorship, and/or publication of this article. This study was supported by Student Research Project Funding Program of Wenzhou Medical University (No. wyx2023101112).

## Author contribution

D.L. and W.D.: conceptualization and design; Y.L., Y.Y., S.Z., and F.N.: methodology; Y.L., Y.Y., and S.Z.: validation; Y.L., Y.Y., S.J., F.N., and J.W.: formal analysis; D.L. and W.D.: investigation; D.L. and W.D.: resources; Y.L., Y.Y., S.J., F.N., J.W., F.C., Y.Z., H.L., H.H., and H.Z.: data curation; Y.L., Y.Y., and S.Z.: writing – original draft preparation; D.L., W.D., Y.L., Y.Y., S.Z., F.N., J.W., F.C., Y.Z., H.L., H.H., H.Z., B.Y., H.C., and X.H.: writing – review and editing; Y.L., Y.Y., and S.Z.: visualization; D.L. and W.D.: supervision; D.L. and W.D.: project administration.

## Conflicts of interest disclosure

The authors declare no conflicts of interest.

## Research registration unique identifying number (UIN)

1. Name of the registry: not applicable.
2. Unique identifying number or registration ID: not applicable.
3. Hyperlink to your specific registration (must be publicly accessible and will be checked): not applicable.

## Guarantor

Not applicable.

## Data availability statement

The data that support the findings of this study are openly available in FinnGen consortium (https://r10.finngen.fi/) and GWAS Catalog (https://www.ebi.ac.uk/gwas/, accession numbers from GCST90001391 to GCST90002121).

## Provenance and peer review

Our paper was not invited.
